# Vascular Health in Adults Born After Using Assisted Reproductive Technologies

**DOI:** 10.1007/s00246-022-03050-4

**Published:** 2022-11-18

**Authors:** Magdalena Langer, Theresa Vilsmaier, Marie Kramer, Franziska Sciuk, Brenda Kolbinger, Pengzhu Li, André Jakob, Nina Rogenhofer, Robert Dalla-Pozza, Christian Thaler, Nikolaus Alexander Haas, Felix Sebastian Oberhoffer

**Affiliations:** 1 Division of Pediatric Cardiology and Intensive Care, University Hospital, LMU Munich, Munich, Germany; 2 Division of Gynecological Endocrinology and Reproductive Medicine, Department of Obstetrics and Gynecology, University Hospital, LMU Munich, Munich, Germany

**Keywords:** Vascular health, Endothelial function, Blood pressure, Arterial stiffness, Assisted reproductive technologies

## Abstract

An increasing number of children are conceived by assisted reproductive technologies (ART). Several studies indicated vascular alterations in ART children. However, limited data is available within the adult ART population. Therefore, this study investigated the overall vascular health of young ART adults in comparison to spontaneously conceived peers. In total, 16 ART subjects and 22 spontaneously conceived peers (22.06 ± 2.21 years vs. 22.00 ± 2.14 years, *p* = 0.194) were enrolled for the assessment of endothelial function, brachial blood pressure, central blood pressure, pulse wave velocity, carotid intima-media thickness, and blood lipids. No significant differences in vascular function were detected between the in vitro fertilization subgroup (*n* = 9), the intracytoplasmic sperm injection subgroup (*n* = 7) and spontaneously conceived peers. This pilot study suggests an unimpaired vascular function in young ART adults. In the future, multi-centric studies with a greater sample size are required to confirm the results of the current study and enable precise cardiovascular risk stratification of the adult ART population.

## Introduction

More than 8 million children have been born with the help of assisted reproductive technologies (ART) worldwide [1]. ART includes in vitro fertilization (IVF) or intracytoplasmic sperm injection (ICSI), with or without cryopreservation of fertilized embryos.

Several studies have raised critical concerns about short- and long-term health consequences of children born via ART [2, 3]. Higher obstetric and perinatal risks like preterm birth, low birth weight, stillbirth, and perinatal mortality were described in children conceived by ART [1, 4–6]. Moreover, an increased risk for birth defects such as congenital heart defects or other major structural malformations was suggested among this cohort [4, 5, 7]. The long-term outcome of subjects conceived by ART has not been addressed yet but might include abnormalities in neurodevelopmental deficits, glucose metabolism, early development of metabolic syndrome and particularly alterations in the cardiovascular system [1, 5, 8].

According to Barker’s 1990 hypothesis of “fetal programming of cardiovascular disease”, adverse health events early in life might be linked to a higher cardiovascular risk at increased age [9]. In addition, environmental exposures in early life might have a long-lasting impact on the later health [1, 6].

Multiple studies were able to demonstrate that children and adolescents conceived by ART show structural and functional alterations of the vascular system [2, 3, 6, 10]. The generalized endothelial dysfunction and increased arterial stiffness shown in pediatric ART cohorts might induce prematurely elevated blood pressure [2, 3, 10]. Early vascular aging in ART conceived subjects could potentially lead to increased cardiovascular morbidity and mortality in adult life.

In contrast, multiple pediatric studies could not demonstrate vascular modifications in ART children compared to spontaneously conceived controls [11, 12]. Moreover, a recent study conducted by Halliday et al. did not detect a significantly increased vascular or cardiometabolic risk in ART adults [13].

Limited data is available on the vascular function of adults who were conceived by ART [13]. Hence, this study aimed to investigate the overall vascular health of young ART adults in comparison to spontaneously conceived peers.

## Materials and Methods

### Ethical Statement

The study was approved on the 27^th^ of December 2020 by the Ethics Committee of the Medical Faculty of the Ludwig Maximilians University Munich (Ethikkommission der Medizinischen Fakultät der Ludwig-Maximilians-Universität München, Pettenkoferstraße 8a, 80336 Munich, Germany; approval number 20-0844) and was performed in accordance with the ethical standards of the Declaration of Helsinki. All participants gave their written informed consent to participate in the present study.

### Study Design and Study Population

The recruitment of ART subjects was conducted in cooperation with the Division of Gynecological Endocrinology and Reproductive Medicine, Department of Obstetrics and Gynecology, University Hospital, LMU Munich (Munich, Germany). One hundred twenty-one couples whose children had been conceived by ART were informed in writing about the ongoing study. Only subjects conceived by IVF or ICSI were included in the present study. Healthy, spontaneously conceived peers were recruited via public calls within the greater Munich area. Study participants were enrolled between May 2021 and March 2022. For the present study, a minimum age of ≥ 18 years was required for participation. Both groups were matched by age and gender. Subjects with medical conditions that could potentially alter the vascular function were excluded from final analysis.

### Medical History, Course of Pregnancy and Birth, Clinical Examination

Study participants were asked about pre-existing health conditions with a strong emphasis on cardiovascular morbidity (e.g., congenital heart disease, arterial hypertension, glucose metabolism disorders, lipid metabolism disorders). Smoking status and medications were recorded. The following information on the course of pregnancy and birth was retrospectively evaluated by screening clinical records and by questioning both parents: birth weight (g), birth height (cm), week of gestation (week), case of multiple pregnancy, maternal age at birth (years), maternal body mass index (BMI, kg/m^2^) at conception, presence of gestational diabetes, maternal blood pressure during pregnancy ≥ 140/90 mmHg, and maternal smoking status during pregnancy. All study participants underwent a complete physical examination.

### Diet Quality

High adherence to the Mediterranean diet was shown to be associated with reduced cardiovascular risk [14–16]. For the assessment of diet quality, the validated 14-Item Mediterranean Diet Assessment Tool established by Martínez-González et al. [14] was translated into German and provided to subjects. According to literature, low adherence to the Mediterranean diet was defined as a score ≤ 7 points and high adherence to the Mediterranean diet if a score ≥ 8 was present [14].

### Physical Activity and Sedentary Behavior

The German version of the Global Physical Activity Questionnaire (GPAQ) developed by the World Health Organization (WHO) was applied to determine the level of physical activity in study participants [17]. Following the recommendations of the WHO, picture cards were presented for each activity domain [17]. Data cleaning was carried out in accordance with GPAQ recommendations [17]. For better comparability of physical activity level, total and recreational Metabolic-Equivalent-(MET)-min were calculated for each study participant [17]. WHO recommendations on physical activity were met if ≥ 600 total MET-min per week were achieved [17]. In addition, the amount of muscle strengthening activities per week as well as the time spent with sedentary activities per day were evaluated.

### Anthropometric Variables

Body height and body weight of each subject was recorded in centimeters and kilograms, respectively. BMI was determined and weight classifications were defined as follows: underweight if BMI < 18.5 kg/m^2^, normal weight if BMI ≥ 18.5 kg/m^2^ but < 25 kg/m^2^, overweight if BMI ≥ 25 kg/m^2^ but < 30 kg/m^2^, obese if BMI ≥ 30 kg/m^2^. The gender-dependent, three-site skinfold protocol of Jackson-Pollock was applied using a skinfold caliper (Harpenden Skinfold Caliper, Baty International, UK) [18, 19]. Body fat percentage (BFP, %) was calculated using the corresponding software (Harpenden Caliper Body Assessment Software, version 1.0.0.17, Baty International, UK).

### Endothelial Function

To assess endothelial function, the EndoPAT^TM^2000 device (Itamar Medical, Israel) was utilized. Study participants were asked to fast ≥ 4 h and abstain from alcohol ≥ 24 h prior to the examination. Special care was taken that the room was quiet and temperature controlled. Before measurement, study participants had to remain in a supine and calm position for at least 15 min. To reduce measurement errors, study participants were asked to keep their fingers still during the entire examination period. The measurement was divided into a 5-min baseline recording period, a 5-min occlusion period and a 5-min post-occlusion period. For occlusion, a cuff was placed on the right upper arm and was inflated between 200 and 300 mmHg to guarantee a complete cessation of the blood flow. After successful examination, the software [version 3.7.2.(2.0)] automatically calculated the reactive hyperemia index (RHI) using the pre- and post-occlusion values.

### Ambulatory Pulse Wave Analysis

Brachial systolic blood pressure (SBP, mmHg), brachial diastolic blood pressure (DBP, mmHg), mean arterial pressure (MAP, mmHg), heart rate (HR, bpm), central SBP (cSBP, mmHg), central DBP (cDBP, mmHg), augmentation index averaged to a heart rate of 75 bpm (AIx@75, %), and pulse wave velocity (PWV, m/s) were assessed using an oscillometric blood pressure device (Mobil-O-Graph®, IEM GmbH, Germany). In adult patients, central blood pressure and PWV assessed by the Mobil-O-Graph® were shown to be accurate and acceptable compared to intra-aortic measurements [20, 21]. Cuff sizes were selected according to right upper arm circumference. Before measurement, study participants had to remain in a supine and calm position for at least 5 min. In accordance with the recommendations of the European Society of Cardiology/European Society of Hypertension, three consecutive ambulatory measurements were performed and averaged to enhance data validity [22]. Elevated blood pressure was present if SBP was ≥ 130 mmHg and/or DBP was ≥ 85 mmHg [22].

### Intima-Media Thickness of the Common Carotid Artery

Both common carotid arteries (CCA) were evaluated by one investigator for all study participants using a Philips iE33 xMatrix or a Philips Epiq 7G ultrasound device (Philips Healthcare, The Netherlands). Following the protocol of Dalla-Pozza et al. [23], both CCA were recorded in a supine position, the neck was extended up to a 45° angle and turned to the opposite side of examination. Using a 3–12 MHz linear array transducer, both CCA were evaluated in long axis view at level of carotid bifurcation. Three consecutive loops were acquired under three-lead ECG tracking. Recorded clips were transferred to a separate workstation (QLAB cardiovascular ultrasound quantification software, version 11.1, Philips Healthcare, The Netherlands) and analyzed by one investigator for all study participants. The carotid intima-media thickness (cIMT, mm) was measured semiautomatically at end-diastole (R wave in ECG) for the right and left side individually. The region of interest was set proximal of the carotid bifurcation and adjusted to 10 mm in length. Three measurements were performed, and an average was calculated for right and left cIMT individually. In addition, the left and right cIMT were averaged.

### Cardiometabolic Risk Profile

Total cholesterol (mg/dL), low-density lipoprotein (LDL) cholesterol (mg/dL), high density lipoprotein (HDL) cholesterol (mg/dL) and lipoprotein(a) [Lp(a), mg/dL] were assessed after a fasting period of ≥ 4 h. The following cut-off values were applied: total cholesterol ≥ 200 mg/dL, LDL cholesterol ≥ 116 mg/dL, HDL cholesterol for females ≤ 48 mg/dL and males ≤ 40 mg/dL, Lp(a) > 30 mg/dL. [24–26]

### Statistical Analysis

Statistical analyses were performed using SPSS 27 (Release Date 2020, IBM SPSS Statistics for Windows, version 27.0.1.0, IBM Corp., Armonk, NY, USA). The Shapiro–Wilk test, histograms and QQ-plots were used to test normality of continuous parameters. Nominal data was compared using the Pearson’s Chi-squared test and Fisher’s exact test. For continuous and normally distributed variables, the analysis of variance (ANOVA) was utilized. Non-normally distributed continuous variables were compared with the Kruskal–Wallis test. Data is given as mean ± SD for normally distributed parameters and as median (minimum/maximum) if non-normally distributed. A *p*-value < 0.05 was considered as statistically significant.

## Results

### Patients’ Characteristics

For the present study, 17 ART subjects and 22 healthy controls were recruited. Within the ART cohort, one subject was excluded from final analysis due to history of T-cell lymphoma. In total, 16 ART subjects (IVF, *n* = 9; ICSI, *n* = 7) and 22 healthy controls were included for final analysis. The groups did not differ significantly in age and gender. Within the IVF group, one subject presented with bicuspid aortic valve, one with questionable history of myocarditis and one with Long QT syndrome. Two subjects took oral contraceptives. Within the ICSI group, one subject suffered from hypothyroidism. One subject was taking oral contraceptives, one thyroid medication and one methylphenidate. Among the control group, six subjects were taking oral contraceptives and one took bisoprolol due to recurrent migraine episodes.

A significant difference in weight classification was shown between all groups. However, after applying a pairwise post hoc test, no significant difference in weight classification was displayed between IVF subjects and controls as well as ICSI subjects and controls. IVF and ICSI subjects demonstrated a significantly higher BFP compared to controls (IVF *p* = 0.047; ICSI *p* = 0.029).

Compared to spontaneously conceived peers, birth weight (*p* = 0.009) and gestational age (*p* = 0.029) were significantly lower in IVF subjects. The number of multiple pregnancies differed significantly between all groups. By applying a pairwise post hoc test, a significantly higher number of multiple pregnancies was revealed in IVF subjects compared to spontaneously conceived peers (*p* < 0.001). In addition, ICSI subjects tended to have a higher number of multiple pregnancies compared to controls (*p* = 0.051). No significant differences in maternal age at birth and maternal BMI at conception were detected between all groups. None of the mothers had gestational diabetes, a blood pressure of ≥ 140/90 mmHg nor smoked during pregnancy.

The Fisher’s exact test showed a significant difference in smoking status among the three groups (*p* = 0.037). However, after applying a pairwise post hoc test, smoking status did not differ between IVF subjects and controls as well as ICSI subjects and controls. There was no significant difference in dietary habits and physical activity level between all three groups.

Absolute values of blood lipids were not significantly different between all groups. A total cholesterol ≥ 200 mg/dL was present in one IVF subject, one ICSI subject and in five controls (*p* = 0.854). A LDL cholesterol ≥ 116 mg/dL was detected in two IVF subjects, two ICSI subjects and six controls (*p* = 1). There were 3 IVF subjects and 3 controls with a decreased HDL cholesterol (*p* = 0.171). A Lp(a) > 30 mg/dL was present in three IVF subjects, one ICSI subject and two controls (*p* = 0.22).

Detailed information on patients’ characteristics is summarized in Table 1.

**Table 1:** Patients’ characteristics in the IVF, ICSI, and control group

Variable	IVF (*n* = 9)	ICSI (*n* = 7)	Control (*n* = 22)	*p*-value
Age (years)	20.97 ± 2.56	21.17 ± 1.86	22.00 ± 2.14	0.427
Female (*n* (%))	7 (77.8)	5 (71.4)	14 (63.6)	0.89
Bodyweight (kg)	67.63 ± 12.21	67.41 ± 10.15	67.61 ± 8.84	1.00
Height (cm)	172.50 ± 8.21	168.37 ± 7.98	175.81 ± 7.83	0.10
BMI (kg/m^2^)	22.16 (16.79/26.32)	23.78 (20.83/27.44)	21.59 (18.93/28.50)	0.17
Weight classification				
Underweight (*n* (%))	1 (11.1)	0 (0)	0 (0)	0.027*
Normal weight (*n* (%))	5 (55.6)	5 (71.4)	21 (95.5)	
Overweight (*n* (%))	3 (33.3)	2 (28.6)	1 (4.5)	
Body fat percentage (%)	29.39 ± 8.12^a^	30.82 ± 5.46^a^	21.57 ± 8.19	0.008**
Course of pregnancy and birth				
Birth weight (g)^1^	2995 (610/3240)^b^	3020(1940/3400)	3420 (1810/4420)	0.007**
Birth height (cm)^2^	51.0 (48.0/54.0)	50.5 (44.0/52.0)	51.00 (43.0/56.0)	0.679
Gestational age (weeks)^1^	37.0 (27.0/39.0)^c^	39.0 (33.0/41.0)	39.0 (33.0/41.0)	0.031*
Maternal age at birth (years)^3^	34.93 ± 2.82	33.53 ± 5.08	32.09 ± 3.34	0.135
Maternal BMI at conception (kg/m^2^)	22.66 ± 2.95	23.69 ± 2.44	21.84 ± 2.18	0.209
Multiple pregnancy (*n* (%))	5 (55.6)^d^	0 (0)	0 (0)	< 0.01**
Smoking, diet quality, and physical activity				
Smoking (*n* (%))	1 (11.1)	2 (28.6)	0 (0)	0.037*
MEDAS	6.56 ± 2.30	5.29 ± 2.93	7.27 ± 1.67	0.10
≥ 600 total MET-min per week (*n* (%))^4^	8 (100)	7 (100)	21 (95.5)	1
≥ 600 recreational MET-min per week (*n* (%))^5^	7 (87.5)	4 (66.7)	18 (81.0)	0.701
Muscle strengthening activities (times/week)^4^	2.0 (0.0/5.5)	1.0 (0.0/ 2.0)	2.0 (0.0/4.0)	0.301
Sedentary behavior (min/day)	383.33 ± 125.30	514.29 ± 176.15	409.09 ± 139.35	0.170
Cardiometabolic risk profile				
Total cholesterol (mg/dL)	171.0 (136/282)	177.0 (143/207)	172.0 (131/243)	0.723
Increased total cholesterol (*n* (%))	1 (11.1)	1 (14.3)	5 (22.7)	0.854
LDL (mg/dL)	94.33 ± 27.17	98.29 ± 15.16	102.27 ± 23.44	0.680
Increased LDL (*n* (%))	2 (22.2)	2 (28.6)	6 (27.3)	1
HDL (mg/dL)	57.0 (35/148)	65.0 (49/107)	61.5 (37/95)	0.773
Decreased HDL (*n* (%))	3 (33.3)	0 (0)	3 (13.6)	0.171
Lp(a) (mg/dL)	5.0 (5/84)	5.0 (5/64)	6.0 (5/98)	0.568
Increased Lp(a) (*n* (%))	3 (33.3)	1 (14.3)	2 (9.1)	0.22

*IVF* In vitro fertilization; *ICSI* Intracytoplasmic sperm injection; *BMI* Body mass index; *MEDAS* Mediterranean diet adherence score; *MET* Metabolic-equivalent; *LDL* Low-density lipoprotein; *HDL* High-density lipoprotein; *Lp(a)* Lipoprotein(a). ^1^18 controls, 8 IVF subjects, and 6 ICSI subjects were included in the analysis. ^2^18 controls, 6 IVF subjects, and 6 ICSI subjects were included in the analysis. ^3^6 ICSI subjects were included in the analysis.^4^8 IVF subjects were included in the analysis. ^5^8 IVF subjects and 6 ICSI subjects were included in the analysis. ^a^IVF differs significantly from control (*p* = 0.047), ICSI differs significantly from control (*p* = 0.029). ^b^IVF differs significantly from control (*p* = 0.009). ^c^IVF differs significantly from control (*p* = 0.029). ^d^IVF differs significantly from control (*p* < 0.001). Data is given as mean ± SD for normally distributed parameters and as median (minimum/maximum) if non-normally distributed. *p < 0.05. **p < 0.01

### Endothelial Function

No significant difference in endothelial function was demonstrated between all groups (Table 2).

**Table 2:** Vascular function in the IVF, ICSI, and control group

Variable	IVF (*n* = 9)	ICSI (*n* = 7)	Control (*n* = 22)	*p*-value
Endothelial function				
RHI^1^	1.96 (1.39/2.89)	2.42 (1.49/3.34)	1.86 (1.37/5.09)	0.567
Ambulatory pulse wave analysis				
SBP (mmHg)	127.81 ± 10.26	120.24 ± 5.42	122.02 ± 7.01	0.104
Elevated SBP (*n* (%))	4 (44.4)	0 (0)	3 (13.6)	0.056
DPB (mmHg)	72.70 ± 7.76	74.14 ± 6.65	73.86 ± 7.89	0.913
Elevated DBP (*n* (%))	1 (11.1)	1 (14.3)	0 (0)	0.171
MAP (mmHg)	98.15 ± 7.84	95.24 ± 4.80	95.77 ± 6.77	0.617
cSBP (mmHg)	113.85 ± 9.17	110.79 ± 5.39	109.85 ± 7.04	0.396
cDBP (mmHg)	74.48 ± 7.98	76.50 ± 7.09	75.29 ± 7.94	0.877
Heart rate (bpm)	65.00 ± 12.83	80.38 ± 16.39	67.48 ± 13.56	0.073
AIx@75 (%)	10.74 ± 10.32	17.29 ± 14.17	11.85 ± 10.72	0.470
PWV (m/s)	5.16 ± 0.40	5.05 ± 0.28	5.13 ± 0.32	0.813
Intima-media thickness of the common carotid artery				
Left cIMT	0.46 (0.40/0.53)	0.43 (0.40/0.48)	0.44 (0.40/0.56)	0.367
Right cIMT^2^	0.44 (0.40/0.49)	0.41 (0.40/0.59)	0.46 (0.40/0.57)	0.264
Averaged cIMT^2^	0.45 (0.40/0.51)	0.44 (0.41/0.50)	0.45 (0.41/0.55)	0.624

*IVF* In vitro fertilization; *ICSI* Intracytoplasmic sperm injection; *RHI* Reactive hyperemia index; *SBP* Systolic blood pressure; *DBP* Diastolic blood pressure; *MAP* Mean arterial pressure; *cSBP* Central systolic blood pressure; *cDBP* Central diastolic blood pressure; *AIx@75* Augmentation index averaged to a heart rate of 75 bpm; *PWV* Pulse wave velocity; *cIMT* Carotid intima-media thickness. ^1^8 IVF subjects were included in the analysis. ^2^21 controls and 5 ICSI subjects were included in the analysis. Data is given as mean ± SD for normally distributed parameters and as median (minimum/maximum) if non-normally distributed

### Ambulatory Pulse Wave Analysis

Ambulatory blood pressure and pulse wave analysis parameters did not show significant differences between groups. Detailed information on ambulatory blood pressure and pulse wave analysis parameters are demonstrated for the IVF, ICSI, and control group in Table 2.

### Intima-Media Thickness of the Common Carotid Artery

Left, right, and averaged cIMT did not display significant differences between IVF, ICSI, and control subjects (Table 2).

## Discussion

To the extent of our knowledge, this is one of the first studies aiming to investigate the overall vascular health of young adults conceived with the help of ART compared to spontaneously conceived peers. In total, 16 ART subjects (IVF, *n* = 9; ICSI, *n* = 7) and 22 age and gender matched controls were included in the present study.

### Vascular Dysfunction and ART: Pathophysiological Considerations

In previous studies, children and adolescents conceived by ART displayed premature vascular aging [2, 3, 10]. This was visualized by a significantly increased PWV and cIMT as well as a significantly reduced flow mediated dilation [2, 3, 10]. As data of Scherrer et al. suggest, arterial hypertension could count as one of the first ART-related signs of vascular dysfunction [10]. In addition, it is thought that the endothelial dysfunction in ART children is not only limited to the systemic circulation but also takes place in pulmonary vessels [2]. The present study was not able to prove the above-mentioned findings in adult ART subjects: All vascular parameters including endothelial function, brachial blood pressure, central blood pressure, PWV, and cIMT did not show significant differences between IVF subjects, ICSI subjects and controls. These results are consistent with previous findings of Halliday et al. [13]. The authors conducted a large cohort study, in which 193 ART subjects and 86 spontaneously conceived peers (mean age: 27.5 ± 2.8 years vs. 27.6 ± 2.6 years, *p* = 0.76) were enrolled [13]. Subjects underwent a detailed cardiovascular evaluation, including the assessment of anthropometric measures, blood pressure, PWV, cIMT, and various metabolic markers [13]. Similarly, to the present study, Halliday et al. demonstrated no evidence of impaired cardiovascular function in the adult ART cohort [13]. Likewise, pediatric studies suggest that children conceived by ART are not at increased cardiovascular risk [11, 12]. A population-based cohort study evaluated the risk of cardiovascular disease or type 2 diabetes in 122,429 ART children and 7,574,685 spontaneously conceived peers from Norway, Sweden, Finland, and Denmark [11]. Interestingly, the authors could not demonstrate a statistically significant difference in the incidence of cardiovascular disease or type 2 diabetes between ART children and spontaneously conceived peers [11]. However, ART children demonstrated a significantly higher risk for obesity [11]. A retrospective Israeli study evaluated hospitalizations due to cardiovascular disease of children born following fertility treatment up to 18 years of age [12]. 2603 IVF subjects, 1721 subjects born as a result of ovulation induction and 237,863 spontaneously conceived peers were included in this study [12]. In comparison to the spontaneously conceived group, no elevated risk of long-term pediatric cardiovascular disease was displayed in the fertility treatment group [12]. The studies of Halliday et al. [13] and Shiloh et al. [12] support the cardiovascular findings of the present study.

Within literature, the following pathophysiological considerations have been reported to impair the vascular health of ART offspring:

While age, gender, diet quality, physical activity level, and blood lipids did not differ significantly between the studied groups, it should be noted that the ART cohort displayed a higher prevalence of smokers and a significantly higher BFP. These cardiovascular risk factors could have potentially negatively influenced the vascular health of the studied ART cohort.

Both Barker’s hypothesis of “fetal programming of cardiovascular disease” and the “developmental origins of adult disease” hypothesis link adverse environmental influences during early human development to an increased risk of non-communicable diseases, such as cardiovascular disease, later in life [9, 27]. “At-risk” pregnancies caused by maternal obesity, maternal overnutrition, gestational weight gain, maternal cardiovascular disease or advanced maternal age might represent such environmental influences [27]. As advanced age and pre-existing cardiovascular diseases negatively affect fertility, individuals with such risk factors might require ART more often. The literature suggests that the prevalence of parental cardiovascular diseases and risk factors, such as hypertension, hyperlipidemia, or diabetes, negatively impact the offspring’s cardiovascular morbidity [28]. Additionally, animal models revealed that advanced maternal age might lead to increased long-term cardiovascular health risks for the offspring [29]. Epidemiological and human studies support these findings [29]. In the present study, neither maternal age at birth, maternal BMI at conception, the prevalence of gestational diabetes nor hypertension during pregnancy differed significantly between the groups.

The ART procedure itself represents an extreme exposure to environmental stimuli for the gamete and embryo, which might result in epigenetic modifications of the DNA potentially causing long lasting alterations of the vascular structure and function [30]. Oxidative stress (OS) is considered to be one possible cause for these epigenetic changes [1]. During the ART procedure, an excess of OS might be present due to a lack of natural antioxidant systems and dysregulation of OS production. In the short-term, OS might cause epigenetic modifications in the DNA [1] and in long-term, studies on animal models propose endothelial dysfunction and hypertension as possible outcomes [1]. In the embryonic development, the cardiovascular system is one of the first to be matured and thus, is proposed to be severely damaged by OS [1]. Increased levels of OS are associated with ART and pregnancies “at risk” (e.g., women of advanced age, obese women, hypertensive disorders of pregnancy, gestational diabetes, and preterm birth) [1]. These risk factors might harm the offspring’s cardiovascular function through increased OS levels [31, 32]. Compared to healthy peers, IVF subjects displayed a lower birthweight, a lower gestational age, and an increased number of multiple pregnancies. Hence, the ART cohort was potentially exposed to higher OS levels early in life.

This pilot study demonstrated that the overall vascular function in ART is not significantly altered in comparison to spontaneously conceived peers. In line with previous findings [13], this study suggests that adults conceived by ART are not necessarily at higher cardiovascular risk. Significant differences in some cardiovascular and perinatal risk factors which might lead to a decreased vascular function at advanced age were displayed. Hence, further studies with larger sample sizes are required to determine potential ART risk groups. An ART follow-up study might be beneficial to assess the extent of increased cardiovascular morbidity and to evaluate the potential process of vascular aging over the life span.

## Limitations

The present study was designed as a single-center study. ART subjects were recruited from one fertility center within Germany. The sample size of the present study can be considered as rather small and heterogeneous. Hence, the assessment of specific cardiovascular risk groups within the ART cohort was not applicable.

Adverse perinatal conditions (e.g., premature birth, multiple pregnancies) were intentionally not defined as exclusion criteria in this study. Preliminary exclusion of such participants was thought to have a positive impact on the ‘actual’ cardiovascular risk profile of the studied ART population as well as substantially restrict the sample size of this single-center study. Hence, further multi-centric studies with a longitudinal study design and larger sample sizes are required for an extensive cardiovascular risk stratification of the adult ART population. Additionally, the ART group studied was at young adult age and vascular changes might only be assessed at a more advanced age.

The results of this study might be attributed to lifestyle habits, parent-related influence, the ART procedure itself or a combination of these factors. Diet quality and physical activity did not differ significantly between all three groups. Even though, no significant impairments of vascular function were detected between all groups, the significantly higher BFP and the higher amount of smokers might have had negative effects on the vascular health of the ART group.

Detailed exploration of maternal cardiovascular risk factors prior to pregnancy and determination of its influence on the offspring’s vascular health seems beneficial. Data on pregnancy and birth was retrospectively evaluated by screening clinical records and by interviewing both parents. For some study participants this data was not complete as clinical records were missing or incompletely filled out by previous medical professionals.

Subjects were required to fast ≥ 4 h and not to drink alcohol ≥ 24 h before study participation. However, a stricter standardization of diet and physical activity of at least ≥ 12 h prior to the vascular measurements should be applied in future studies. In literature, a reduction in SBP and DBP after exercise is defined as post-exercise hypotension [33]. The duration of this phenomenon is described to last between 2 and 13 h post-exercise [33]. Therefore, future studies should consider this potential cofounder and adjust their study design accordingly.

As the investigators of the current study were not blinded, the results may have been influenced, in particular the evaluation of cIMT. In contrast, various vascular parameters (e.g., RHI, blood pressure) were assessed automatically by devices.

## Conclusion

This pilot study investigated the vascular health of 16 young ART adults. In contrast to previous studies, the present study was not able to detect any significant differences in vascular function between ART subjects and spontaneously conceived peers. The results of this study might weaken previous ART health concerns which have been raised over the past years. In the future, multi-centric studies with a greater sample size are required to confirm the results of the current study and enable precise cardiovascular risk stratification of the adult ART population.
